# Supplementary material for: Comparative Performance Testing of Respirator versus Surgical Mask Using a Water Droplet Spray Model
Source: Int J Environ Res Public Health. 2021 Feb 8;18(4):1599. doi: 10.3390/ijerph18041599 (PMC7915861; doi:10.3390/ijerph18041599)
Supplement: Supplementary file 1 [file ijerph-18-01599-s001.pdf]

Supplementary material

Comparative performance testing of respirator versus surgical mask using a water droplet spray model

Heiman F.L. Wertheim*[1,2], Paul T.J. Scheepers*[3], Henk Jan Holterman[4], Steven Teerenstra[3], Liesbeth Martens[1,2], Martijn de Groot[5], Andreas Voss[1,6], Joost Hopman[1,2]

Method

The particle size distribution of the jet spray was measured at distances of 30 cm and 60 cm from the nebulizer outlet by phase-doppler anemometry using a Phase Doppler Particle Analyzer (PDPA) system (TSI, Shoreview, MN, USA). The nebulizer is shown in **Figure S1**. In **Figure S2** the measurement set-up of nebulizer and PDPA is presented.

Results

<u>Particle size distribution</u>

Figure S3 provides the mean number and volume particle size distributions for both 30 and 60 cm distances based on the PDPA measurements.

<u>Total inward leakage (TIL)</u>

The TIL data obtained at 30 and 60 cm are provided as means ± standard deviations (**Table S1)** and as point estimates with 95% confidence intervals (95% CI) and the p-values of the comparisons reported in the main text (**Table S2)**.

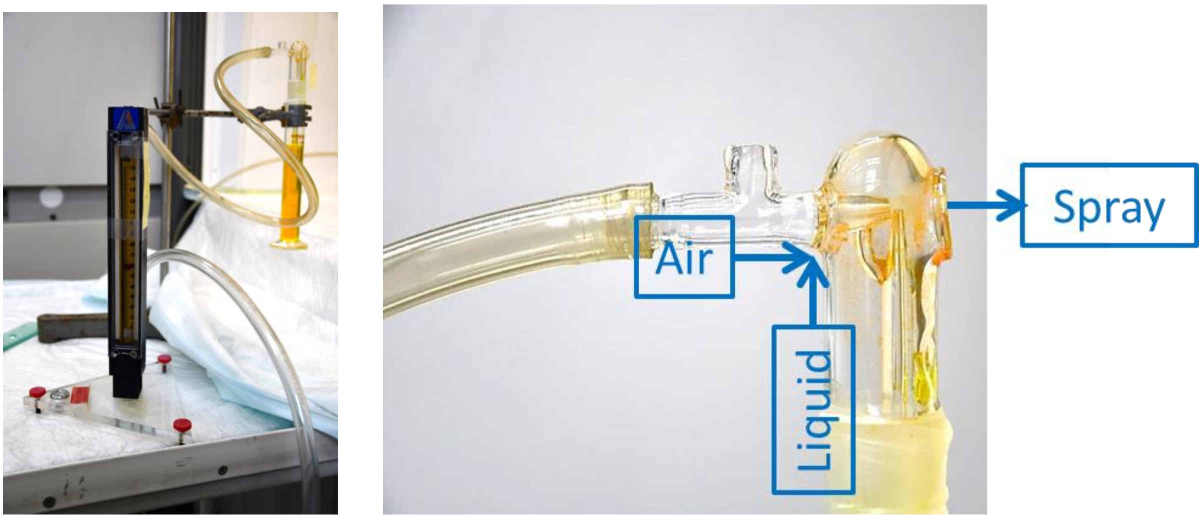

(a) **(b)**

Figure S1. Cross-flow nebulizer with rotameter (a) and close up of top with liquid and air entry and spray outlet (b). To activate the nebulizer, the opening at the top must be closed.

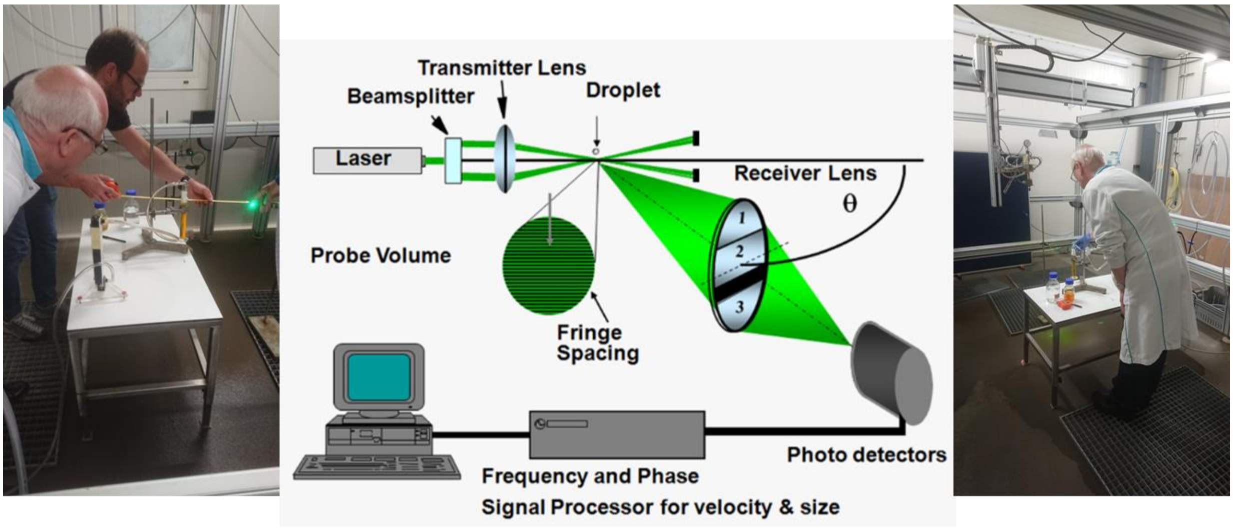

(a) **(b)** **(c)**

Figure S2. Set-up for particle size distribution measurement: adjusting the distance between nebulizer and measuring point (a); set-up of the PDPA principle (Source: Berg et al. 2005) (b); using the nebulizer in the spraying experiment (c).

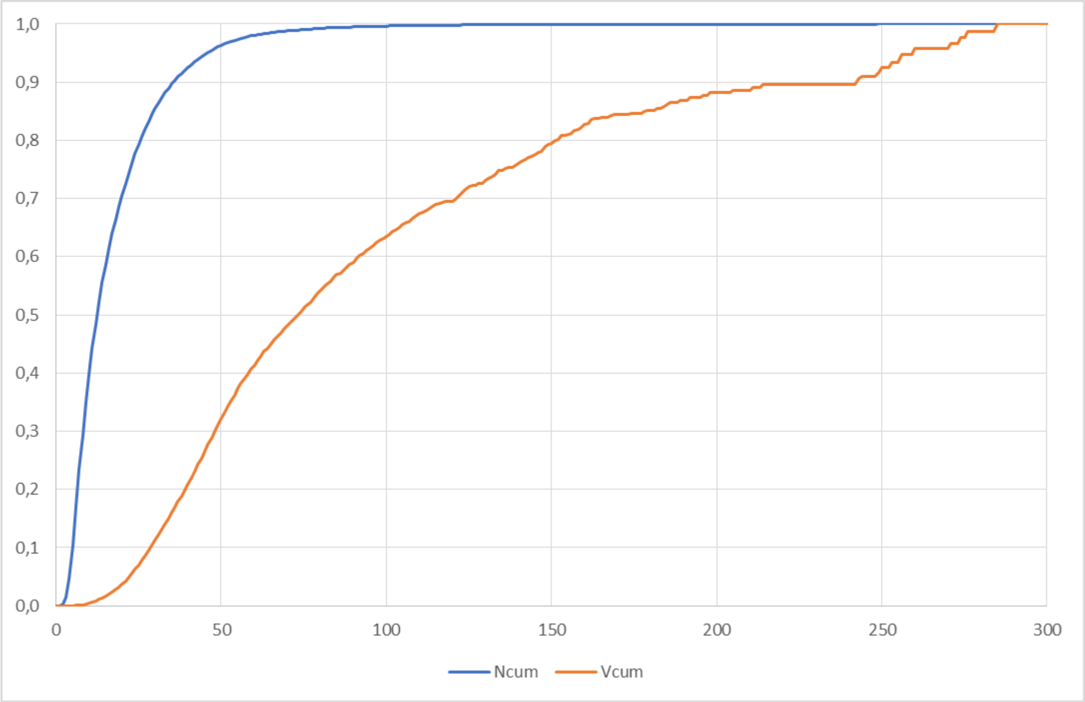

.

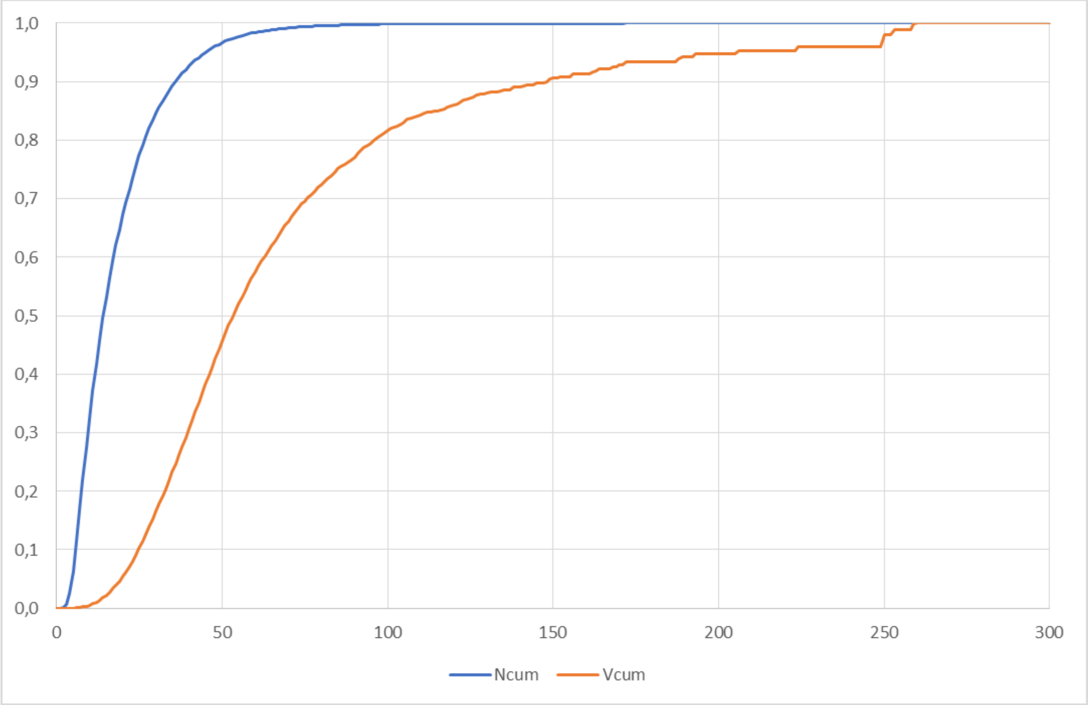

Figure S3. Cumulative number and volume distributions for jet spray of the solution of flurorescein in MilliQ water at 30 cm (a) and 60 cm (b) each based on four replicates.

Table S1. TIL based on retrieved fluorescein (ng).

Test material	30 cm[a]		60 cm[b]	
	N	Mean ± sd	N	Mean ± sd
FFP2	16	1,320.7 ± 290.6	5	1,643.3 ± 124.7
FFP2 + face shield	10	1,194.8 ± 466.3	--	--[c]
Surgical mask	10	1,086.4 ± 247.6	5	1,287.2 ± 188.8
Surgical mask + face shield	10	906.3 ± 462.2	--	--[c]
Face shield only	11	1,423.8 ± 198,7	5	1,855.3 ± 370.7

[a] Sample of 15 min with single spray challenge during 10 sec at a distance of 30 cm; [b] Sample of 15 min with first spray challenge (10 s) and after 5 min followed by a second spray challenge (10 s) both at a distance of 60 cm; [c] Not tested**Table S2.** Point estimates with 95%-confidence intervals

Distance	Comparison	Estimate	Lower	Upper	p-value
30 cm	FFP2 -- surgical mask	234.34	20.0194	448.67	0.0327
	FFP2 + face shield – surgical mask + face shield	288.43	-128.19	705.04	0.1707
	FFP2 + face shield -- FFP2	-125.94	-455.80	203.92	0.4471
	Surgical mask + face shield -- surgical mask	-180.02	-512.74	152.69	0.2826
	FFP2 + face shield -- face shield only	-229.06	-688.77	230.64	0.3220
	Surgical mask + face shield -- face shield only	-517.49	-975.53	-59.4560	0.0276
60 cm	FFP2 -- surgical mask	356.10	135.58	576.62	0.0042
	surgical mask -- face shield only	-568.04	-973.44	-162.64	0.0100
	FFP2 -- face shield only	-211.95	-593.08	169.19	0.2490

Reference

Berg T, Deppe J, Michaelis D, Voges H, Wissel S (2005) Comparison of particle size and velocity investigations in sprays carried out by means of different measurement technicques. Proceedings Institute for Liquid Atomization and Spray Systems, August 27-September, 2006, Kyoto, Japan, Paper ID ICLASS06-151. http://www.ilasseurope.org/ICLASS/ICLASS2006/DATA/PDF/F3-01-151.pdf (Accessed 09-10-2020)
